# Successful Pregnancy in a Case of Behçet's Disease after Treatment with Prednisolone

**DOI:** 10.1155/2020/8862651

**Published:** 2020-10-15

**Authors:** Shogo Nishii, Koushi Yamaguchi, Mitsuyoshi Amita, Takakazu Saito, Hidekazu Saito, Akihiko Sekizawa

**Affiliations:** ^1^Department of Obstetrics and Gynecology, School of Medicine, Showa University, Tokyo 142-8555, Japan; ^2^Center of Maternal-Fetal, Neonatal and Reproductive Medicine, National Center for Child Health and Development, Tokyo 157-8535, Japan; ^3^Division of Reproductive Medicine, National Center for Child Health and Development, Tokyo 157-8535, Japan

## Abstract

A 34-year-old woman (gravida 1, para 0) visited the Division of Reproductive Medicine/National Center for Child Health and Development due to infertility; she had also been suffering from incompletely treated genital ulcers and stomatitis for 10 years. This case was diagnosed as an incomplete-type Behçet's disease (BD) at the Department of Maternal-Fetal Biology/National Center for Child Health and Development. Since no apparent abnormality was found in the general infertility test, artificial insemination with the husband's semen (AIH) was performed for the patient with unexplained infertility, which failed. However, after treating BD with prednisolone, chronic inflammation (stomatitis and genital ulcer) and immunological abnormalities (Th2 and NK cell activity) improved, and conception was possible by AIH. Thus, prednisolone administration may have induced immune tolerance in the patient with BD, which may have contributed to the success of AIH.

## 1. Introduction

Behçet's disease (BD) is a common inflammatory disease in Japan, and the peak age of onset is in the 30s [1]. Even though the onset mechanism of this disease is not clear, it results from the disruption of the immune system by multiple factors. Among them, some external environmental conditions act on specific internal genetic factors [2]. Due to genetic mutations in several receptors, a large number of inflammatory cytokines is produced, and the sensitivity of the receptors that recognize them is increased, leading to the accumulation of other lymphocytes and neutrophils in the lesion and establishment of a BD immune response. More importantly, this immune response in women with BD can affect fertility, as high risks of miscarriage and stillbirth were observed in the study by Orgul et al. [3]. They suggested that BD-associated inflammatory processes at the maternofetal interface were associated with the occurrence of these complications. However, the mechanism by which the immune system affects the fertility of females with BD has not yet been elucidated.

Here, we report a case in which a woman diagnosed with BD conceived following artificial insemination of husband's semen (AIH) after chronic inflammation and immune conditions were improved by the administration of an immunosuppressant. We additionally provide details on the immune mechanism associated with infertility in this patient.

## 2. Case Presentation

**Figure 1:**
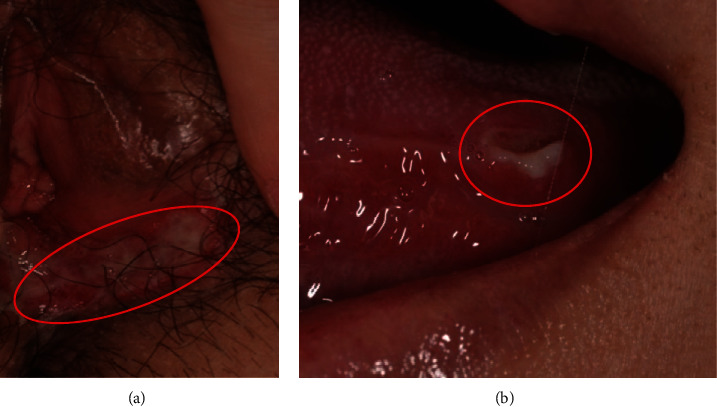
(a) A genital ulcer identified on the inside of the left labia minora (red circle). (b) A 5 mm aphthous ulcer (red circle) on the left side of the tongue.

A 34-year-old nulliparous woman (gravida 1, para 0) of 158 cm height and 53 kg weight had no allergies and no family history of the disease. She had been suffering from recurrent oral and genital ulcers that were not properly treated for 10 years. Moreover, she had a history of early miscarriage and had been suffering from infertility for two years after that unfavorable event. For this reason, the patient visited the Infertility Center and later consulted the Department of Maternal-Fetal Biology. Moreover, the genital ulcers and the stomatitis got worse. Her physical examination revealed a 5 mm aphthous ulcer on the left side of the tongue. In addition, a genital ulcer was identified on the inside of the left labia minora (Figures 1(a) and 1(b)). The patient's diagnosis was an incomplete-type BD, with the main symptoms being recurrent genital and oral ulcerations (herpetiform) and skin lesions. Furthermore, no arthritis, gastrointestinal, vascular, or ocular lesions were observed. Laboratory analyses revealed that total protein (TP) [8.2 g/dL (normal range, 6.5–8.2)], C-reactive protein (CRP) [0.44 mg/dL (normal range, 0.00–0.30)], immunoglobulin G (IgG) levels [2266 mg/dL (normal range, 870–1700)], white blood cell count (WBC) [9300/*μ*L (normal range, 4000–9000)], and 50% hemolytic complement activity (CH50) [57.3 U/mL (normal range, 30–45)] were higher than the normal levels, while iron (Fe) levels [29 *μ*g/dL (normal range, 48–154)] were lower than normal. The percentages of Th1 (interferon (IFN)*γ*+/IL-4-/CD4+) cells, Th2 (IFN-*γ*-/IL-4+/CD4+) cells, and activated natural killer (NK) cells were 10.1%, 4.2%, and 56% (normal range, 18–40), respectively. Other laboratory findings related to miscarriage, such as antiphospholipid syndrome (anti-CL IgM Ab, anti-PE IgG Ab, and anti-PE IgM Ab), autoimmune disease (ANA and anti-DNA Ab), and disorders of blood coagulation (protein S activity, protein C activity, and factor XII activity), were all negative.

**Figure 2:**
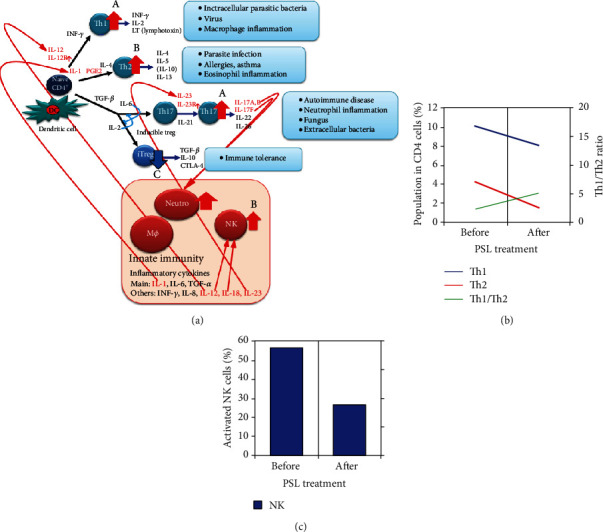
(a, A) Enhanced sensitivity of IL-12R and IL-23R leads to the differentiation of naïve CD4+ cells to Th1 and Th17 cells (red arrows). (B) IL-1, produced mainly by macrophages, increases the number of Th2 cells via PGE2, and IL-12 and IL-18-activate NK cells (red arrows). (C) When Th17 induction is strong, the induction of regulatory T cells (Tregs) reduces (blue arrow), and immune tolerance cannot be reached. MØ: macrophage; Neutro: neutrophil. (b) Th1 cell fraction decreased from 10.1% to 8.1% after prednisolone (PSL) treatment; Th2 cell fraction decreased from 4.2% to 1.6%; and the Th1 to Th2 ratio increased from 2.4 to 5.1. (c) NK cell activity decreased from 56% to 26% after PSL treatment. The patient was administered 10 mg/day PSL for 4 weeks, which improved the immune status.

All the general fertility tests, including the husband's semen analysis, performed at the Department of Reproductive Medicine indicated no apparent abnormalities. Therefore, we performed AIH through ovulation induction with clomiphene citrate for treating unexplained infertility. After the failure of first AIH, the patient was treated with 10 mg/day prednisolone (PSL) for 4 weeks, which improved the genital ulcer, chronic inflammation (TP, platelet (PLT), CRP, CH50, Fe, and IgG), and immune status (Th2 and NK activity) (Table 1, Figures 2(b) and 2(c)). Pregnancy was established at the second AIH attempt after PSL administration. PSL was maintained at 8 mg/day during the course of the pregnancy, and no mucosal lesions were observed. At 38 weeks and 4 days of gestation, a healthy baby boy weighing 3090 g was vaginally delivered.

## 3. Discussion

Chronic inflammation signs, increased percentages of Th2 cells, and enhanced NK cell activity were observed in this patient. Additionally, although the percentages of Th17 cells, which play a central role in BD, could not be analyzed due to restriction of the clinical laboratory, we expected that they would increase, while those of regulatory T cells (Tregs) would decrease because of the Th17/Treg balance in the process of differentiation of naïve CD4+ cells. These parameters returned to normal, and the patient conceived following the administration of PSL.

Embryo implantation and successful pregnancy are only established under maternal immune tolerance [4]. Therefore, an abnormal number of T cells and the increased NK cell activity present in this case, indicative of chronic inflammation, significantly interfered with the implantation. Medawar first pointed out this immunological discrepancy in 1953 [5]. Lédée et al. reported that the immune system was either activated or suppressed in cases of implantation failure [6]. In general, T cells are divided into Th1, which produce IL-2 and IFN-*γ*, and Th2 cells, which produce IL-4, IL-5, IL-10, and IL-13 (Figure 2(a), A and B). Th1 cells are involved in cellular immunity, and when they become dominant, they attack the fetus, leading to miscarriage and pregnancy-induced hypertension [7]. In contrast, Th2 dominance is required for successful implantation and pregnancy. Recurrent implantation failure and pregnancy loss were negatively correlated with Th1 cell numbers [8]. In addition, NK cells increased in number at the decidua in a case of chromosome-normal abortion [9].

BD is a polysymptomatic, chronic, and recurrent systemic vasculitis [10] associated with mutations in the IL-12R and IL-23R genes and decreased IL-10 protein expression [11, 12]. Enhanced sensitivity of IL-12R and IL-23R leads to the differentiation of naïve CD4+ cells into Th1 and Th17 cells. Furthermore, IL-23R activates Th17 cells (Figure 2(a), A), which are deeply involved in the defense against infection, such as the elimination of extracellular bacteria, neutrophil inflammation, and autoimmune pathogenesis. BD mainly induces a Th17-centered neutrophil inflammation, and its activity is significantly correlated with Th17 serum levels [13]. Naïve CD4+ cells differentiate into both Th17 and Treg cells in the presence of TGF-*β*. The strong induction of Th17 cells by IL-6 suppressed the production of Treg cells by IL-2, which disturbed the immune tolerance (Figure 2(a), C). Furthermore, the suppression of IL-10 expression increased Th1 cell proliferation [14]. Thus, various inflammatory cytokines released by activated T cells may act on other lymphocytes and neutrophils in a complex manner to establish the inflammatory and immune response characteristic to BD and lead to infertility.

In this case, high numbers of Th2 cells were possibly evoked by activated Th17 cells via IL-17A and B due to a strong neutrophil inflammation. When innate immunity is activated, many inflammatory cytokines are produced, such as IL-1, which is derived from macrophages and induces differentiation towards Th2 cells via PGE2, and IL-12 and IL-18, which activate NK cells (Figure 2(a), B). PSL administration reduced the number of both Th1 and Th2 cells and the NK cell activity, which are important cells for fetal rejection. The decrease in Th17 cells after treatment with PSL led to a normal induction of Treg cells, which induced immune tolerance. Furthermore, decreased IgG, TP, CRP, CH50, and PLT levels and higher Fe amounts improved chronic inflammatory condition. Additionally, the patient recovered from genital ulcer and recurrent aphthous lesions of the oral mucosa.

Infertility rate did not increase in properly treated BD patients [15]. PSL administration to the patient improved the chronic inflammation (Table 1) and decreased the Th2 cell fraction and NK cell activity (Figures 2(b) and 2(c)), thus promoting immune tolerance, which probably helped in conception. It is already known that PSL downregulates both Th1 and Th2 responses [16]. The pathogenesis of autoimmune diseases such as BD is largely unknown, and its association with infertility is still under investigation. According to the results in the presented case, the immunological condition of the patient and its association with infertility need a more detailed examination.

The immune abnormalities associated with BD development are not completely known. In our case, they hindered embryo implantation. However, pregnancy was established after PSL administration, as it led to an improved immunological status of the patient with BD.

## Figures and Tables

**Table 1:** Levels of chronic inflammatory parameters before and after treatment.

	PSL treatment	
	Before	After
TP (g/dL)	8.2	7.7
Alb (g/dL)	4.4	4.2
WBC (/*μ*L)	9300	13620
PLT (/*μ*L)	33.5	27.7
CRP (mg/dL)	0.44	0.11
CH50 (U/mL)	75.3	50.8
Fe (*μ*g/dL)	28	96
IgG (mg/dL)	2266	1692
IgA (mg/dL)	168	155
IgM (mg/dL)	168	177

The patient was treated with 10 mg/day prednisolone (PSL) for 4 weeks, which improved the genital ulcer and chronic inflammation signs such as TP, PLT, CRP, CH50, Fe, and IgG. TP: total protein; Alb: albumin; WBC: white blood cells; PLT: platelet; CRP: C-reactive protein; CH50: 50% hemolytic complement activity; Fe: iron; IgG: immunoglobulin G; IgA: immunoglobulin A; IgM: immunoglobulin M.
